# Environmental Impoverishment, Aging, and Reduction in Mastication Affect Mouse Innate Repertoire to Explore Novel Environments and to Assess Risk

**DOI:** 10.3389/fnins.2019.00107

**Published:** 2019-03-14

**Authors:** Fabíola de Carvalho Chaves de Siqueira Mendes, Luisa Taynah Vasconcelos Barbosa da Paixão, Cristovam Wanderley Picanço Diniz, Marcia Consentino Kronka Sosthenes

**Affiliations:** ^1^Laboratório de Investigações em Neurodegeneração e Infecção, Instituto de Ciências Biológicas, Hospital Universitário João de Barros Barreto, Universidade Federal do Pará, Belém, Brazil; ^2^Curso de Medicina, Centro Universitário do Estado do Pará, Belém, Brazil

**Keywords:** reduced mastication, environmental changes, aging, locomotor and exploratory activities, anxiety-like behavior

## Abstract

Studies indicate that inhibition of adequate masticatory function, due to soft diet, occlusal disharmony, or molar losses affects the cognitive behavior of rodents. However, no study has tested the effects on new environments exploration and risk assessment coupled with a combination of masticatory function rehabilitation and environmental enrichment. In the present report, we tested the hypothesis that age, environment, and masticatory changes may interact and alter exploratory patterns of locomotor activity and mice preferences in an open field (OF) arena. As OF arenas are widely used to measure anxiety-like behavior in rats and mice. We examined in an open arena, the exploratory and locomotor activities of mature (6-month-old; 6M), late mature (12-month-old; 12M), and aged (18-month-old; 18M) mice, subjected to distinct masticatory regimens and environments. Three different regimens of masticatory activity were used: continuous normal mastication with hard pellets (HD); normal mastication followed by reduced mastication with equal periods of pellets followed by soft powder – HD/SD; or rehabilitated masticatory activity with equal periods of HD, followed by powder, followed by pellets – HD/SD/HD). Under each diet regimen, half of the individuals were raised in standard cages [impoverished environment (IE)] and the other half in enriched cages [enriched environment (EE)]. Animals behavior on the open field (OF) task were recorded by webcam and analyzed with Any Maze software (Stöelting). The locomotor and exploratory activities in OF task declined with age, and this was particularly evident in 18M HD EE mice. Although all groups kept their preference by the peripheral zone, the outcomes were significantly influenced by interactions between environment, age, and diet. Independent of diet regime, 6M young mice maintained in an EE where voluntary exercise apparatus is available, revealed significant less body weight than all other groups. Although body weight differences were minimized as age progressed, 18M EE group revealed intragroup significant influence of diet regimens. We suggest that long life environmental enrichment reduces the tendency to avoid open/lit spaces (OF) and this is particularly influenced by masticatory activity. These measurements may be useful in discussions of anxiety-related tasks.

## Introduction

Mastication seems to contribute to maintain body weight within normal limits, and a soft diet has been associated with obesity in murine model (Desmarchelier et al., 2013). The latter outcome appears to be associated with significant changes in hypothalamic synaptic input organization and gliosis (Horvath et al., 2010). This type of diet-induced obesity progressively alters cognition and mouse performance in the elevated plus maze (EPM) task (André et al., 2014). In addition, it has been reported that inhibition of adequate masticatory function, due soft-diet feeding, occlusal disharmony or molar losses, affected mice cognitive behavior (Onozuka et al., 2000; Yamamoto and Hirayama, 2001; Tsutsui et al., 2007; Kubo et al., 2010; Ono et al., 2010; Frota De Almeida et al., 2012; Ekuni et al., 2013; Mendes et al., 2013; Sakatani et al., 2013; Nose-Ishibashi et al., 2014; Utsugi et al., 2014; Pang et al., 2015; Takeda et al., 2016; Aguirre Siancas, 2017). Furthermore, loss of molars early in life (Kawahata et al., 2014) or soft diet consumption (Fukushima-Nakayama et al., 2017) caused loss impaired hippocampal-dependent recognition memory and induced a lateralized preference of object location in recognition tasks (Kawahata et al., 2014). In a previous report, we mimicked an active and a sedentary-life styles in murine model and tested the effects of age, environment and diet regimes on spatial memory, using Morris water-maze task (Mendes et al., 2013). We found that an enriched environment (EE) and masticatory activity rehabilitation recover spatial memory decline in aged mice.

However, only a few reports have investigated the effects of a soft diet (Nose-Ishibashi et al., 2014) and aging on sedentary-like and active murine models and no studies have tested the combined effects of masticatory rehabilitation and environmental enrichment on innate repertoire to explore novel environments and to assess risk. In the present report, we tested the hypothesis that age, environment, and masticatory changes may interact and alter mice exploratory patterns of locomotor activity and preferences in an OF arena. Open arenas are widely used for measuring anxiety-like behavior in mice and rats. In these tasks, when animals explore an unfamiliar area, they remain close to the walls; this preference is taken as an indication of fear-induced anxiety (Lalonde and Strazielle, 2008; Ennaceur, 2014). Hiding behavior may contribute to avoiding attack and predation, and it may be included in the repertoire of animal survival instincts. The species-specific hiding response in mice appears to lie at the root of their natural preference for unlit and protected spaces. Thus, the OF tests are based on the natural tendencies in mice to avoid open/lit areas and to spontaneously explore unfamiliar areas (Komada et al., 2008). Thus, animals appear to innately avoid open and/or lit spaces in the central area of the OF (Ennaceur, 2014). Mouse preference for the safety of the peripheral zone of the OF may reflect this adaptive response; for a recent review (see Ennaceur, 2014).

Thus, we focused on these preferred regions to compare mouse behaviors and to detect potential differential effects of diet regimes, age and environmental changes on mouse locomotor and exploratory activities.

To that end we examined outcomes of mice exploratory and locomotor activities in an OF. Tested individuals were under influence of different masticatory regimens, environments and age. Mature (6-month-old; 6M), late mature (12-month-old; 12M), and aged (18-month-old; 18M) mice were maintained either in standard laboratory or enriched cages. Masticatory regimens included: continuous normal mastication with hard pellets (HD); normal mastication followed by reduced mastication, with equal periods of pellets followed by soft powder; and rehabilitated masticatory activity with equal periods of HD, followed by powder, followed by pellets.

## Materials and Methods

Female albino Swiss mice were maintained in animal housing, in accordance with the guidelines published by the National Institutes of Health (Guide for the Care and Use of Laboratory Animals). The experimental protocol was tested and approved prior to study initiation by the Ethics Committee on Experimental Animal Research (from the Institute of Biological Sciences, Federal University of Pará, Brazil, CEPAE-UFPA: BIO223-14).

### Age, Diets, Environments, and Body Weight

Masticatory activity was manipulated with different diets. Each age group was subjected to either a diet of HD, which required substantial chewing, or alternation with powder soft food (SD), which required little chewing. Masticatory rehabilitation was employed by feeding different sequences of HD and SD to mice.

**FIGURE 1:**
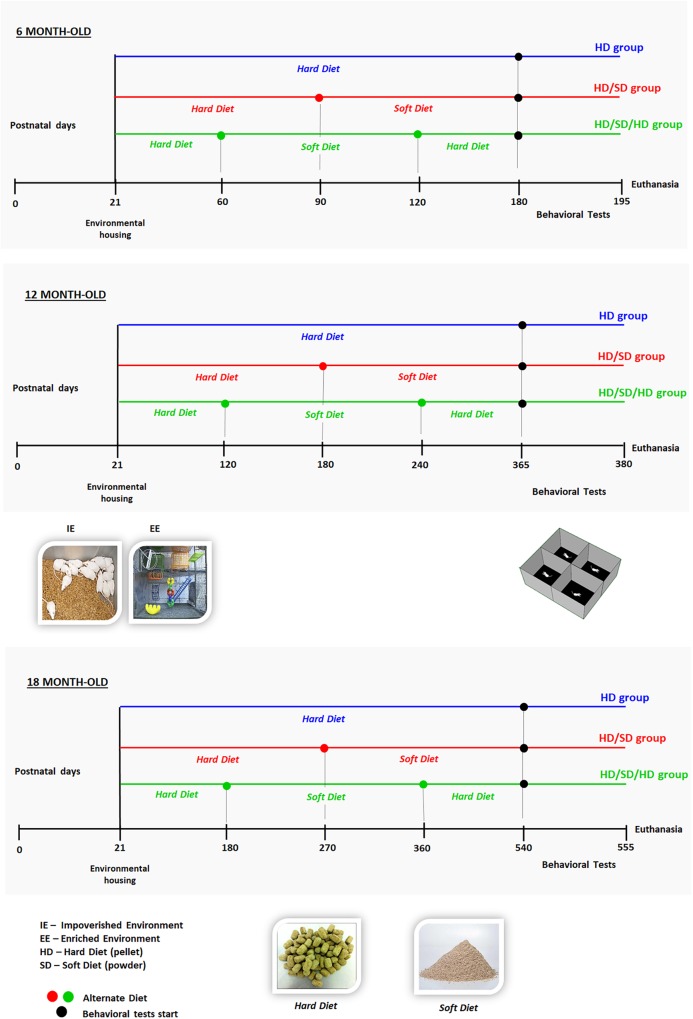
Experimental setup and timeline. Time is indicated non-linearly, increasing from left to right, from birth (day 0) to euthanasia. Mice were housed in either an impoverished or enriched environment (IE or EE; images of environments are shown in the *middle panel, left*) from postnatal days 21 to 180 (*top*, 6 months old; 6M), 365 (*in the middle*, 12 months old; 12M), or 540 (*from bottom*, 18 months old; 18M). All animals were submitted to the open field (OF) tasks (test apparatus is shown in the *middle panel, right*); test was performed at the points indicated with filled circles on the corresponding timelines. Timelines are color-coded to indicate the following diet regimes: HD (blue): pellet diet; HD/SD (red): alternating pellet and powder diets, switched at 3, 6, or 9 months, respectively, for 6M, 12M, or 18M mice; and HD/SD/HD (green): alternating pellet, powder, and pellet diets, switched every 2, 4, or 6 months, respectively, for 6M, 12M, or 18M mice. *Bottom panel:* images show the hard pellets (HD) and soft powder (SD).

The mice were raised either in sedentary-like or active conditions (Figure 1). The impoverished environment (IE) comprised plastic cages (32 cm × 45 cm × 16.5 cm) limited to chopped rice straw on the floor (no equipment or toys) covered with metal grids. Each IE cage housed eight individuals per age group: young (6M), or middle-aged (12M), or aged (18M) mice. The EE consisted of two-level wire cages (100 cm × 50 cm × 100 cm) equipped with rod bridges, tunnels, ropes, toys, and running wheels. Toys were composed of different forms of plastic, wood, and metal of different colors, and they were changed periodically. Each EE cage housed 12 mice per age group: 6M or 12M, or 18M (the total number of animals per experimental group is described in Table 1). Water and food were delivered to the top and bottom levels, respectively. This arrangement obliged mice to move from one floor to the other, when they wanted to drink and eat. All animals had free access to food and water. All mice were raised in a controlled room temperature (23 ± 1°C) and a 12-h light–dark cycle. To detect potential influences of diet regimes and environments on body weight, all animals were weighed at the beginning of experiment and at the end of each time window (6M, 12M, and 18M).

**Table 1:** Number of animals for each group.

Environment	Age	Diet	Number of animals
IE	6M	HD	8
		HD/SD	8
		HD/SD/HD	8
	12M	HD	8
		HD/SD	8
		HD/SD/HD	8
	18M	HD	8
		HD/SD	8
		HD/SD/HD	8
EE	6M	HD	12
		HD/SD	12
		HD/SD/HD	12
	12M	HD	12
		HD/SD	12
		HD/SD/HD	12
	18M	HD	12
		HD/SD	12
		HD/SD/HD	12

*In the table are presented the environments [impoverished environment (IE); enriched environment (EE)], diets (HD, HD/SD and HD/SD/HD), and the age 6M, 12M, and 18M (6, 12, and 18 months old, respectively).*

### Behavioral Tests

All mice were behaviorally assessed only once according to age (6, 12, or 18 months). All groups were tested in an OF before euthanasia. The apparatus and test protocol were slightly modified from previous report (Rodgers and Johnson, 1995).

The OF apparatus consisted of a gray polyvinyl chloride box (30 cm × 30 cm × 40 cm), with a floor divided into central and peripheral regions of equal areas (Figure 4). Each animal was placed at the center of the apparatus for 5 min. All experiments were carried out following the same protocol which included also the tests at the same time of day, conditions of luminosity (3 cd/m^2^) and handling of the animals.

A video camera connected to a computer was placed one meter above the OF. Each training session was recorded for later analysis with Any-Maze software (Stöelting), and this allowed to record the precise position of each mouse throughout OF test. The following parameters were analyzed: distance traveled, minute-by-minute (m), time spent in the peripheral zone (seconds) and in the corners of the test apparatus. The times were also expressed as the percentage of the total time. After each session, the OF apparatus was cleaned with 70% ethanol.

### Statistical Analysis of Behavioral Changes

No blinding procedure was applied for behavioral tests since video records and software do not allow researcher influence. It is also important to mention that all experiments were performed according to the protocol described in previous session. Significant differences between groups with respect to the behavioral tasks were evaluated with three-way ANOVA and Tukey’s *post hoc* test honestly significant difference. We also investigated the influences of age, diet regime, and environment on behavioral outcomes; differences between groups were considered significant with a 95% confidence level cutoff (*p* < 0.05). Analyses were performed with EzAnova or BioEstat 5.0 (Ayres et al., 2007) software. To apply three-way factorial ANOVA to the results of OF test, we used the percentage of time spent in peripheral area, as a function of the total time test. One-way ANOVA and the Tukey’s *post hoc* test honestly significant difference were used to analyze significant differences in distance traveled along each minute of OF test. Differences between groups were considered significant at the 95% confidence level (*p* < 0.05).

## Results

### Intergroup Analysis

We investigated the effects of masticatory activity changes, environment, and age on the pattern of exploratory activity in the OF test. Thus, three-way ANOVA analysis applied to time spent in peripheral zone revealed that it was significantly influenced by environment [*F*_(1,90)_ = 4.55; *p* < 0.036] and diet [*F*_(2,90)_ = 8.9; *p* < 0.0003], but not by age [*F*_(2,90)_ = 3.07; *p* < 0.0512]. The combination of environment and diet did not show a significant interaction [*F*_(2,90)_ = 0.76; *p* < 0.470].

However, all other variables acting together showed that OF test results were significantly influenced by interactions between the environment and age [*F*_(2,90)_ = 11.3; *p* < 0.000042]; age and diet [*F*_(4,90)_ = 6.00; *p* < 0.00025]; and environment, age, and diet [*F*_(4,90)_ = 9.72; *p* < 0.000001]. These variables (environment and age; age and diet; and environment, age, and diet) interacted either subtractive or additively to influence OF results.

### Intragroup Analysis

**FIGURE 2:**
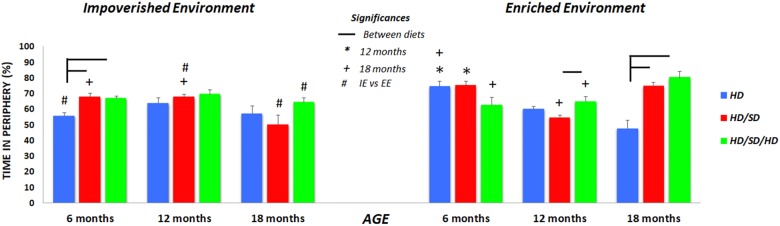
The percentages of time spent in the periphery of the OF. (^∗^) Significantly different from 12 months; (+) significantly different from 18 months; (#) significant difference between impoverished (IE) and enhanced environments (EE); line connectors indicate significant differences between diets. HD (blue) = hard diet; HD/SD (red) = hard diet followed by soft diet; HD/SD/HD (green) = hard diet followed by soft diet followed by hard diet.

**FIGURE 3:**
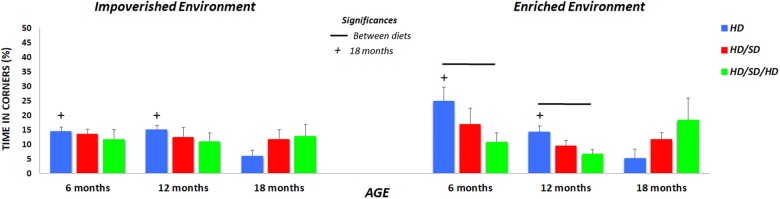
Quantification of the time spent in the corners of the OF. Times are shown for mice raised in **(left)** impoverished or **(right)** enhanced environments. (+) Significantly different from the 18-month group; lines indicate significant differences between diets; HD (blue) = hard diet; HD/SD (red) = hard diet followed by soft diet; HD/SD/HD (green) = hard diet followed by soft diet followed by hard diet.

In the OF task, the HD individuals of 6M mice group raised in IE, spent less time in the periphery as compared to mice of the same age under all other diet regimes maintained at the same environmental condition (Figure 2). Related to 6M mice raised in EE, the influence of diet regimes was limited, because the environmental stimulation seems to increase the preference of the control group (HD EE 6M) by the peripheral zone, minimizing the observed differences between diets among the IE animals. In Figure 3, this preference of the HD EE 6M by the periphery and especially by the corners of the apparatus are illustrated.

Among 12M and 18M groups maintained in IE, no significant effect of diet changes is observed, but it is interesting to highlight that only the HD/SD IE group reduces their preference for the peripheral zone when the animals of 6M and 12M are compared with those of 18M (Figure 2). Another particular difference between diet regimens was observed in the old mice group from enriched environment (18M EE). Mice fed with hard diet (HD – control group), spent less time in periphery zone than mice from all other diets. Interestingly, as age progresses, mice tend to decrease their preference for the peripheral zone, and this behavior is more evident among animals kept in EE and with no change in masticatory activity.

In the case of animals raised in IE, this age effect seems to be present only in the HD/SD group as previously commented. In addition, groups that underwent alteration of masticatory activity also appeared to be more sensitive to environmental influences (IE vs. EE in HD/SD 12M and 18M; IE vs. EE in HD/SD/HD 18M).

Because independent of experimental variables, the experimental groups spent at least half of test time in OF peripheral zone, we investigated potential preferences for selected points of this zone such as its corners. We speculate that corners may be associated with less risk. Thus, Figure 3 illustrates the percentage of test time spent at the corners of the apparatus. Note that reduction of time spent in corners was greater related to aging, and this is evident in HD group, independent of the environment where mice were raised.

More detailed comparisons (mean, standard error, *F*-values, and *p*-values) were performed for the different experimental groups (see Supplementary Table 1) to determine the effects of diet regime, environment, and age on OF test outcomes.

**FIGURE 4:**
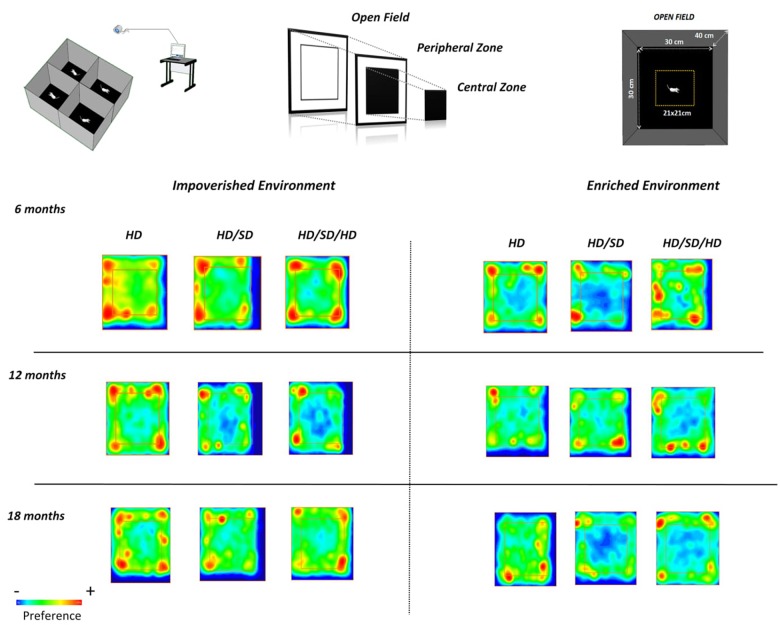
Graphic representations of preferred areas in the OF test. (*Top row*, *left*) Experimental setup and video capture system for recording mouse behavior in the OF test; (*top middle and right*) the peripheral and central regions are equal in area. (*Lower panels*) Time spent in all areas by mice raised in impoverished (*left*) or enriched (*right*) environments, at different ages (*rows*). The square region more central (internal) delineates the central (external) and peripheral areas of the OF. Red, yellow, and blue indicate high, intermediate, and low preferences, respectively. Diet regimes are indicated at the top: HD = hard diet; HD/SD = hard diet followed by soft diet; HD/SD/HD = hard diet followed by soft diet followed by hard diet.

Figure 4 demonstrate experimental groups preferences for selected OF regions. In this figure, red color indicates higher preferences and blue one, a smaller preference. Note that mice rarely visited the central region of the OF test, independent of age, environment, or diet regimen. However, as discussed earlier, note that the control group 18M maintained in enriched environment (HD EE 18M) avoid the peripheral zone and corners of the OF apparatus while chewing alteration groups avoid the center of the arena (HD/SD and HD/SD/HD at EE 18M).

**FIGURE 5:**
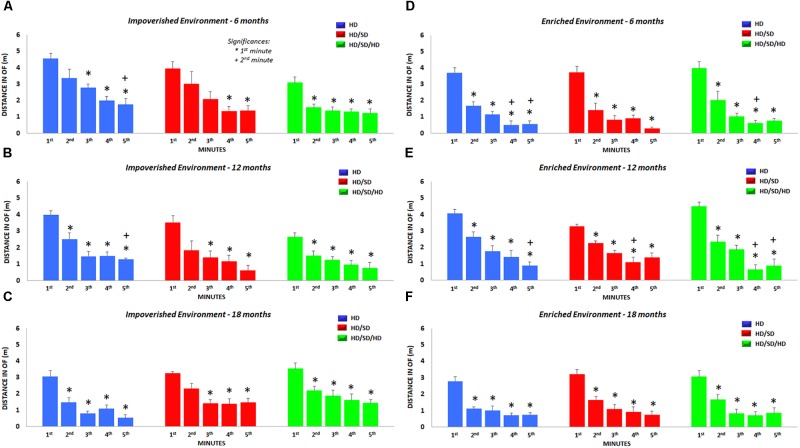
Distance traveled in the OF test. Total distance per minute traveled during the OF test is shown for each diet regime (color-coded), age (*top row*: 6M, *middle row*: 12M, and *bottom row*: 18M), and environment (*left*: impoverished and *right*: enriched environment). (^∗^) Significantly different from the 1^st^ minute; (+) significantly different from the 2^nd^ minute; HD (blue) = hard diet; HD/SD (red) = hard diet followed by soft diet; HD/SD/HD (green) = hard diet followed by soft diet followed by hard diet.

We also analyzed the total distance traveled in each minute of test, independent of spatial location occupied in the OF (Figure 5) and found significant reductions in the locomotor and exploratory activities over the 5 min. These minute-by-minute analysis revealed that traveled distance decrease as time progresses, and that the time-dependent reduction was accentuated by aging and environmental enrichment.

More detailed comparisons (mean, standard error, *F*-values, and *p*-values) were performed for the different experimental groups (Supplementary Table 2) to determine the effects of diet regime, environment, and age on OF outcomes.

### Body Weight

At the beginning of the experiment no statistically significant difference was found related to weights of the animals in the different groups.

#### Intergroup Analysis

A three-way ANOVA of body weights recorded on the day before euthanasia of each group revealed that body weight was significant influenced by environment [*F*_(1,90)_ = 32.4, *p* < 0.000001], age [*F*_(2,90)_ = 3.45, *p* < 0.036], and diet [*F*_(2,90)_ = 14.4, *p* < 0.000004]. Significant interactions between environment and diet [*F*_(2,90)_ = 3.62, *p* < 0.03], and between environment and age [*F*_(2,90)_ = 15.1, *p* < 0.000002] were observed. There was not significant interaction between age and diet [*F*_(4.90)_ = 2.29, *p* < 0.066] or between environment, age, and diet [*F*_(4,90)_ = 0.201, *p* < 0.937].

#### Intragroup Analysis

**FIGURE 6:**
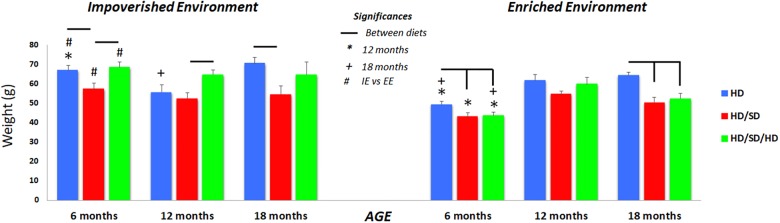
Quantification of the weight body at the end of each age analyzed. (^∗^) Significantly different from 12 months; (+) significantly different from 18 months; (#) significant difference between impoverished (IE) and enhanced environments (EE); line connectors indicate significant differences between diets. HD (blue) = hard diet; HD/SD (red) = hard diet followed by soft diet; HD/SD/HD (green) = hard diet followed by soft diet followed by hard diet.

Within the 6M and 12M age group, created in IE, mice rehabilitated to normal masticatory activity (HD/SD/HD) showed significant higher body weights than that mice with reduced masticatory activity (HD/SD group) (Figure 6). This increasing condition was not observed in the groups created in EE at the same time windows. However, HD weighed significantly more than HD/SD in IE and EE at 6M, and more than HD/SD/HD in EE at the same age. The same occurred at 18 months of age.

Comparing the environments, the EE 6M was associated with a significant body weight reduction compared to IE mice, independent of diet regime, but no significant differences were detected between IE and EE mice at 12 or 18 months.

Related to different ages, the weight gain of animals in the 12M group, regardless of the diet regime, was significantly higher compared to 6M, as well as the weight gain at 18M, and this was especially noted in animals raised in EE.

More detailed comparisons (mean, standard error, *F*-values, and *p*-values) were performed for the different experimental groups to determine the effects of diet regime, environment, and age on weight outcomes and are depicted on Supplementary Table 3.

## Discussion

In a previous report, we demonstrated that a 24-h, complex EE had differential effects on performance in episodic-like and water maze memory tests, among young and aged female albino Swiss mice (Diniz et al., 2010). We also showed that imposing reduced masticatory activity, by offering a powder diet to mice previously fed a pellet (HD/SD), caused impaired spatial learning, but a diet that imposed masticatory rehabilitation (HD/SD/HD) reversed the impairments in aged mice (Mendes et al., 2013).

Part of these effects can be explained by studies that evidence that masticatory dysfunction, sustainably activates the Hypothalamic-Hypophysis-Adrenal (H-H-A) axis increasing blood stream glucocorticoid levels. The continuous increase in circulating glucocorticoids in turn interrupts H-H-A axis negative feedback system, further increasing secretion of glucorticoids (Azuma et al., 2017). However, neurogenesis in dentate gyrus, which is critical to hippocampal-mediated modulation in H-H-A axis also appears to be impaired by masticatory dysfunction causing cognitive deficits which are dependent on morphofunctional integrity of the hippocampus (Snyder et al., 2011).

In this context, high levels of glucorticoids could still be modulating anxiety-like or fear behavior. The act of hiding to avoid attacks and predation ends up being an innate strategy to guarantee animal survival and this logic follows OF tests in which apparatus they have a natural tendency to avoid open areas (Komada et al., 2008).

The EPM, although widely used in these cases, seems to suffer undesirable influence from environmental manipulation that may increase or decrease locomotor activity generating false positive or negative test results (Dawson and Tricklebank, 1995). Exposure to a new environment immediately prior to EPM, for example, increases motor activity in maze increasing time spent in open arms (File and Wardill, 1975a,b; Pellow et al., 1985).

In our laboratory, mice are often subjected to a sequence of tasks, which include OF testing, object recognition, followed by EPM and forced swimming. In these cases, it is common to perform each task on separate occasions. However, in face of such evidence of pre-exposure to another environment influencing the EPM results we assume that careful consideration should be given when designing experiments using EPM.

Therefore, we tested the hypothesis that a combination of masticatory rehabilitation and EE would minimize the impaired exploratory and locomotor behavioral changes induced by a combination of IE and reduced masticatory activity. We demonstrated that all mice, independent of the masticatory condition, environment, and age, exhibited a similar temporal organization of their spatial horizontal exploratory activity in the OF task. Nevertheless, we found that the EE, aging, and normal masticatory activity (HD), interacting with each other, reduced the innate tendency to avoid open/lit spaces in the OF task. We also demonstrated that contrasting diet regimes, designed to reduce or rehabilitate masticatory activity, had differential effects at different time windows.

In the OF test sedentary young adults from IE with reduced masticatory activity or rehabilitation (HD/SD and HD/SD/HD IE 6M) showed a higher preference for the periphery of the OF than control animals from similar environment (HD IE 6M). This result may suggest that the preference for the peripheral zone observed in the groups with masticatory alterations may be associated with potential stress due to masticatory changes, either associated with masticatory reduction or rehabilitation. This result is in line with previous report, showing that the total distance of locomotion was significantly higher for animals fed with soft diet previously fed with hard diet, suggesting that the shift to powdered diet may affect the responsiveness of mice exposed to new environments (Nose-Ishibashi et al., 2014). Indeed, it has been proposed that early in life masticatory manipulation may increase vulnerability for mental disorders (Nose-Ishibashi et al., 2014).

Horizontal locomotor exploration comprises part of the innate repertoire used by animals to explore novel environments and to assess risk in the wild (Augustsson and Meyerson, 2004; Ennaceur, 2014). It has been suggested that this strategy arises from the drives to avoid and to explore a perceived threatening stimulus (Crusio, 2001). In the present study, we promptly recognized this stereotypical behavior, when mice explored the OF (Lister, 1990; Kalueff et al., 2006; Ennaceur, 2014).

With exposure to predator odors, a previous report (Sampedro-Piquero et al., 2016), demonstrated that EE could reduce anxiety in aged Wistar rats, when confronted with cat odor. That finding was indicated by a time reduction in freezing behavior. Similar EE effects were observed in aged Wistar rats on an elevated-zero maze task (Sampedro-Piquero et al., 2013, 2014).

Although a controversial issue due to methodological differences, species and strains used in the tests, it is important to highlight that EE may contribute either to reduce or increase anxiety-like behavior and depression (Greenwood et al., 2003; Burghardt et al., 2004; Van Hoomissen et al., 2004; Duman et al., 2008; Leasure and Jones, 2008).

In the present report, the HD EE 18M group compared with HD EE 6M individuals, exhibited a reduction in anxiety-like behavior, suggesting that open arena adaptive behavior in this group may be associate with a continuous non-aversive EE which includes normal chewing, voluntary exercise, and visuospatial stimulation as part of the necessary repertoire to adapt to an open arena with reduced stress level (Dupire et al., 2013). In agreement, our findings revealed that, as compared with individuals from IE, animals raised in EE, decreased traveled distance more rapidly along the test, and this was particularly significant to 18M mice.

An alternative view is that the environmental enrichment may increase stress level due to the weekly change in the location or substitution of old by new toys (Larsson et al., 2002). This chronic low level of stress may adapt old mice from EE to face novelty (Konkle et al., 2010).

Allied to this, as in the present work the tests were performed only in females, it is important to discuss some possible influences of estrous cycle. We have seen that estrus phases and respective ovarian hormones oscillations in rats may be mediating defensive behaviors (Blume et al., 2017), which are associated with fear and anxiety (Graham and Scott, 2018; Pentkowski et al., 2018), including neuronal changes in amygdala nuclei (such as the lateral and basal). However, despite evidence on these influences, it is considered that estrous cycle may be affected by animal housing conditions too. Andrade et al. (2002) reported that females from overpopulation cages, without males, appear to exhibit a phase designated as anestrous characterized by the absence of estrus cycles. In our case, the estrous cycle phases were not investigated in the animals and since we only worked with females and only females inhabited our laboratory, we expect little influence of the cycle on the behavioral results detected. However, because we did not measure potential influences of hormones on behavior, future studies should take this as a limitation of the present study. Despite of this, it is consistent to say that the older mice was depleted of estrogenic protection. Thus, we reasoned that at least part of the behavioral changes may be related to the estropause.

When analyzed the differences in the body weight of the animals, as compared with controls HD or rehabilitated HD/SD/HD mice, the body weight from HD/SD mice, fed proportionally longer with soft diet revealed significant reduction of their body weight. This finding does not agree with previous data demonstrating long term soft diet-induced obesity (Nojima et al., 2006; Desmarchelier et al., 2013; André et al., 2014). Coherently, we found no correlation between preferred zone of the OF and body weight as previously described in rats with weight losses induced by gastrectomy as compared with rats fed with hypercaloric diets (Himel et al., 2018).

Notwithstanding, it is important to recognize that the environmental enrichment influenced the weight gain. Indeed, independently of the diet regime at 6M all EE groups weighed less than those from IE. In contrast to animals from IE, EE seemed to enhance body weight differences between 6M, 12M, and 18M mice reducing age related metabolic differences.

Therefore, we conclude that changes in masticatory activity, influence the pattern of exploration by zones in the OF test and environmental impoverishment seems to enhance the effects of aging, increasing the preference for the peripheral zone of OF by the animals that not have undergone chewing alteration. Finally, the environmental enrichment also influences mice weight gain, but this is not correlated with spatial pattern of explored zones in the OF.

## Author Contributions

FCCSM participated in the development and methodological design, collection and treatment of data, analysis and interpretation of data and writing. LTVBP participated in the collection and processing of data. CWPD participated in the development and methodological design, supervision, analysis and interpretation of data and writing. MCKS participated in the development and methodological design, supervision, analysis and interpretation of data and writing.

## Conflict of Interest Statement

The authors declare that the research was conducted in the absence of any commercial or financial relationships that could be construed as a potential conflict of interest.
