# Supplementary material for: Environmental Impoverishment, Aging, and Reduction in Mastication Affect Mouse Innate Repertoire to Explore Novel Environments and to Assess Risk
Source: Front Neurosci. 2019 Mar 14;13:107. doi: 10.3389/fnins.2019.00107 (PMC6427831; doi:10.3389/fnins.2019.00107)
Supplement: Supplementary file 1 [file Presentation_1.pdf]

Table 1: Representation of the mean and respective standard error values of the total distance traveled in meters, minute by minute, during the 5 minutes of open field test for the various experimental groups in the different diet regimes (HD, HD/SD and HD/SD/HD), age (6M, 12M and 18M) in an impoverished environment (IE).

	**DISTANCE (m): mean ± standard error**				
	**Impoverished Environment**				
**GROUPS**	**1st minute**	**2nd minute**	**3th minute**	**4th minute**	**5th minute**
HD – 6M	4.54 ± 0.33	3.35 ± 0.56	2.77 ± 0.24*	1.97 ± 0.29*	1.74 ±0,37*$^{+}$
HD/SD – 6M	3.94 ± 0.42	3.01 ± 0.76	2.06 ± 0.46	1.33 ± 0.30*	1.37 ± 0.31*
HD/SD/HD – 6M	3.09 ± 0.35	1.57 ± 0.21*	1.38 ± 0.23*	1.31 ± 0.17*	1.21 ± 0.27*
HD – 12M	3.97 ± 0.26	2.51 ± 0.38*	1.48 ± 0.29*	1.50 ± 0.22*	1.28 ± 0.10*
HD/SD – 12M	3.51 ± 0.43	1.83 ± 0.43	1.38 ± 0.43*	1.16 ± 0.43*	0.61 ± 0.43*
HD/SD/HD – 12M	2.63 ± 0.25	1.51 ± 0.30*	1.25 ± 0.21*	0.96 ± 0.26*	0.76 ± 0.34*
HD – 18M	3.03 ± 0.38	1.47 ± 0.28*	0.78 ± 0.14*	1.08 ± 0.23*	0.52 ± 0.19*
HD/SD – 18M	3.24 ± 0.11	2.31 ± 0.31	1.41 ± 0.23*	1.36 ± 0.33*	1.44 ± 0.27*

HD/SD/HD – 18M	3.54 ± 0.36	2.20 ± 0.26*	1.87 ± 0.35*	1.59 ± 0.38*	1.42 ± 0.23*

(*) Indicates significant intra-group difference with the first minute of the test; (+) intra-group significant difference simultaneous with the first and second test minutes.

Table 2: Representation of the mean and respective standard error values of the total distance traveled in meters, minute by minute, during the 5 minutes of open field test for the various experimental groups in the different diet regimes (HD, HD/SD and HD/SD/HD), age (6M, 12M and 18M) in an enriched environment (EE).

	**DISTANCE (m): mean ± standard error**				
	**Enriched Environment**				
**GROUPS**	**1st minute**	**2nd minute**	**3th minute**	**4th minute**	**5th minute**
HD – 6M	3.70 ± 0.30	1.68 ± 0.25*	1.16 ± 0.17*	0.49 ± 0.25*+	0.56 ± 0.20*+
HD/SD – 6M	3.71 ± 0.40	1.40 ± 0.43*	0.81 ± 0.25*	0.89 ± 0.21*	0.27 ± 0.11*
HD/SD/HD – 6M	3.97 ± 0.42	2.02 ± 0.55*	1.01 ± 0.23*	0.62 ± 0.16*+	0.75 ± 0.16*
HD – 12M	4.05 ± 0.26	2.61 ± 0.32*	1.74 ± 0.36*	1.37 ± 0.43*	0.86 ± 0.25*+
HD/SD – 12M	3.28 ± 0.13	2.23 ± 0.15*	1.63 ± 0.18*	1.07 ± 0.33*+	1.37 ± 0.30*
HD/SD/HD – 12M	4.49 ± 0.25	2.34 ± 0.39*	1.86 ± 0.28*	0.64 ± 0.28*+	0.87 ± 0.42*+
HD – 18M	2.76 ± 0.31	1.11 ± 0.10*	1.00 ± 0.28*	0.70 ± 0.15*	0.72 ± 0.17*
HD/SD – 18M	3.19 ± 0.31	1.64 ± 0.23*	1.09 ± 0.29*	0.91 ± 0.32*	0.73 ± 0.24*

HD/SD/HD – 18M	3.06 ± 0.37	1.68 ± 0.30*	0.82 ± 0.24*	0.70 ± 0.24*	0.85 ± 0.33*

(*) Indicates significant intra-group difference with the first minute of the test; (+) intra-group significant difference simultaneous with the first and second test minutes.

Table 3: Representation of the values obtained after the one-way ANOVA in the post-test of Tukey (q) and p-value (p) significant for the distance traveled (m), minute by minute of test, in the open field for the groups (HD, HD/SD, HD/SD/ HD), age (6M, 12M and 18M) in an impoverished (IE) and enriched environment (EE).

GROUPS		ANOVA-one way	1st *vs* 2nd (minute)	1st *vs* 3th (minute)	1st *vs* 4th (minute)	1st *vs* 5th (minute)	2nd *vs* 4th (minute)	2nd *vs* 5th (minute)
HD 6M	**IE**	$F_{(4,25)} = 9.1151$ $p = 0.0002$	-	$q_{(10)} = 4.7253$ $p < 0.05$	$q_{(10)} = 6.8697$ $p < 0.01$	$q_{(10)} = 2.7992$ $p < 0.01$	-	$q_{(10)} = 4.2848$ $p < 0.02$
	**EE**	$F_{(4,25)} = 30.5695$ $p < 0.0001$	$q_{(10)} = 8.4839$ $p < 0.01$	$q_{(10)} = 10.715$ $p < 0.01$	$q_{(10)} = 13.5215$ $p < 0.01$	$q_{(10)} = 13.2194$ $p < 0.01$	$q_{(10)} = 5.0375$ $p < 0.05$	$q_{(10)} = 4.7355$ $p < 0.05$
HD/SD 6M	**IE**	$F_{(4,25)} = 5.4621$ $p = 0.003$	-	-	$q_{(10)} = 5.4363$ $p < 0.01$	$q_{(10)} = 5.342$ $p < 0.01$	-	-
	**EE**	$F_{(4,25)} = 19.4155$ $p < 0.0001$	$q_{(10)} = 7.5731$ $p < 0.01$	$q_{(10)} = 9.5196$ $p < 0.01$	$q_{(10)} = 9.2597$ $p < 0.01$	$q_{(10)} = 11.2729$ $p < 0.01$	-	-
HD/SD/HD 6M	**IE**	$F_{(4,25)} = 9.448$ $p = 0.0002$	$q_{(10)} = 5.9893$ $p < 0.01$	$q_{(10)} = 6.7303$ $p < 0.01$	$q_{(10)} = 6.9904$ $p < 0.01$	$q_{(10)} = 7.3971$ $p < 0.01$	-	-
	**EE**	$F_{(4,25)} = 16.8077$ $p < 0.0001$	$q_{(10)} = 5.7118$ $p < 0.01$	$q_{(10)} = 8.6927$ $p < 0.01$	$q_{(10)} = 9.8386$ $p < 0.01$	$q_{(10)} = 9.4477$ $p < 0.01$	-	-
HD 12 M	**IE**	$F_{(4,25)} = 17.928$ $p < 0.0001$	$q_{(10)} = 5.4764$ $p < 0.01$	$q_{(10)} = 9.3724$ $p < 0.01$	$q_{(10)} = 9.2668$ $p < 0.01$	$q_{(10)} = 10.121$ $p < 0.01$	-	$q_{(10)} = 4.6446$ $p < 0.01$
	**EE**	$F_{(4,25)} = 14.404$ $p < 0.0001$	$q_{(10)} = 4.3518$ $p < 0.05$	$q_{(10)} = 7.0149$ $p < 0.01$	$q_{(10)} = 8.1216$ $p < 0.01$	$q_{(10)} = 9.6755$ $p < 0.01$	-	$q_{(10)} = 5.3237$ $p < 0.01$

HD/SD 12 M	**IE**	$F_{(4,25)} = 6.6058$ $p < 0.0012$	-	$q_{(10)} = 4.9511$ $p < 0.05$	$q_{(10)} = 5.4645$ $p < 0.01$	$q_{(10)} = 6.7575$ $p < 0.01$	-	-
	**EE**	$F_{(4,25)} = 13.95$ $p < 0.0001$	$q_{(10)} = 4.467$ $p < 0.05$	$q_{(10)} = 7.072$ $p < 0.01$	$q_{(10)} = 9.4348$ $p < 0.01$	$q_{(10)} = 8.1573$ $p < 0.01$	$q_{(10)} = 4.9678$ $p < 0.01$	
HD/SD/HD 12 M	**IE**	$F_{(4,25)} = 22.519$ $p < 0.0009$	-	$q_{(10)} = 4.931$ $p < 0.05$	$q_{(10)} = 6.033$ $p < 0.01$	$q_{(10)} = 6.7367$ $p < 0.01$	-	-
	**EE**	$F_{(4,25)} = 21.254$ $p < 0.0001$	$q_{(10)} = 6.4674$ $p < 0.01$	$q_{(10)} = 7.9073$ $p < 0.01$	$q_{(10)} = 11.537$ $p < 0.01$	$q_{(10)} = 10.868$ $p < 0.01$	$q_{(10)} = 5.0695$ $p < 0.05$	$q_{(10)} = 4.4002$ $p < 0.05$
HD 18 M	**IE**	$F_{(4,25)} = 14.64$ $p < 0.0001$	$q_{(10)} = 6.0426$ $p < 0.01$	$q_{(10)} = 8.6932$ $p < 0.01$	$q_{(10)} = 7.5354$ $p < 0.01$	$q_{(10)} = 9.7051$ $p < 0.01$	-	-
	**EE**	$F_{(4,25)} = 15.894$ $p < 0.0001$	$q_{(10)} = 7.6515$ $p < 0.01$	$q_{(10)} = 8.1971$ $p < 0.01$	$q_{(10)} = 9.5698$ $p < 0.01$	$q_{(10)} = 9.4729$ $p < 0.01$	-	-
HDSD 18 M	**IE**	$F_{(4,25)} = 9.89$ $p < 0.0002$	-	$q_{(10)} = 7.024$ $p < 0.01$	$q_{(10)} = 7.2156$ $p < 0.01$	$q_{(10)} = 6.8892$ $p < 0.01$	-	-
	**EE**	$F_{(4,25)} = 12.621$ $p < 0.0001$	$q_{(10)} = 5.5302$ $p < 0.01$	$q_{(10)} = 7.4723$ $p < 0.01$	$q_{(10)} = 8.1109$ $p < 0.01$	$q_{(10)} = 8.7644$ $p < 0.01$	-	-
HDSDHD 18 M	**IE**	$F_{(4,25)} = 6.9$ $p < 0.0009$	-	$q_{(10)} = 5.1966$ $p < 0.01$	$q_{(10)} = 6.0487$ $p < 0.01$	$q_{(10)} = 6.5969$ $p < 0.01$	-	-

	**EE**	$F_{(4,25)} = 10.939$ $p < 0.0001$	$q_{(10)} = 4.6062$ $p < 0.05$	$q_{(10)} = 7.4353$ $p < 0.01$	$q_{(10)} = 7.8482$ $p < 0.01$	$q_{(10)} = 7.3601$ $p < 0.01$	-	-
